# Lycorine Induces Mitochondria-Dependent Apoptosis in Hepatoblastoma HepG2 Cells Through ROCK1 Activation

**DOI:** 10.3389/fphar.2019.00651

**Published:** 2019-06-06

**Authors:** Wu-yi Liu, Qin Tang, Qian Zhang, Chang-peng Hu, Jing-bin Huang, Fang-fang Sheng, Ya-li Liu, Min Zhou, Wen-jing Lai, Guo-bing Li, Rong Zhang

**Affiliations:** Department of Pharmacy, The Second Affiliated Hospital, Army Medical University, Chongqing, China

**Keywords:** lycorine, mitochondria, apoptosis, HepG2 cells, ROCK1

## Abstract

Lycorine, a naturally occurring compound extracted from the *Amaryllidaceae* plant family, has been reported to exhibit antitumor activity in various cancer cell types. In the present study, we investigated the molecular mechanisms underlying lycorine-induced apoptosis in hepatoblastoma HepG2 cells. We found that lycorine induced mitochondria-dependent apoptosis in HepG2 cells accompanied by mitochondrial permeability transition pore (mPTP) opening, mitochondrial membrane potential (MMP) loss, adenosine triphosphate (ATP) depletion, Ca^2+^ and cytochrome c (Cyto C) release, as well as caspase activation. Furthermore, we found Rho associated coiled-coil containing protein kinase 1 (ROCK1) cleavage/activation played a critical role in lycorine-induced mitochondrial apoptosis. In addition, the ROCK inhibitor Y-27632 was employed, and we found that co-treatment with Y-27632 attenuated lycorine-induced mitochondrial injury and cell apoptosis. Meanwhile, an *in vivo* study revealed that lycorine inhibited tumor growth and induced apoptosis in a HepG2 xenograft mouse model in association with ROCK1 activation. Taken together, all these findings suggested that lycorine induced mitochondria-dependent apoptosis through ROCK1 activation in HepG2 cells, and this may be a theoretical basis for lycorine’s anticancer effects.

## Introduction

Rho-associated protein kinase (ROCK), a well-characterized Rho effector, is reported to be involved in many cellular processes, including actin dynamics, cell adhesion and migration, apoptosis, proliferation, and metabolism (Leung et al., 1996; Matsui et al., 1996; Totsukawa et al., 2000; Shi and Wei, 2007). In recent years, high expression of ROCK1 has been reported in several human cancers and often correlated with poor survival (Liu, 2011). Moreover, studies have reported that the inhibition of ROCK1 increases survival in various animal disease models (Whatcott et al., 2017). Furthermore, ROCK1 has been developed as a potential therapeutic target for diseases like neurological disorders, cardiovascular diseases, and cancers (Akagi et al., 2014; Hartmann et al., 2015; Henderson et al., 2016).

Apoptosis, which occurs in the process of cellular ageing, plays an important role in the homeostasis and development in normal tissues (Lockshin and Williams, 1965). Moreover, induction of apoptosis during carcinogenesis is considered to effectively attenuate the progression of cancers (Kerr and Searle, 1972). Current studies have reported two main apoptotic pathways: the intrinsic or mitochondrial pathway and the extrinsic or death receptor pathway. As we all know, mitochondria, the primary energy source for cells, play a significant role in cell metabolism (Gogvadze et al., 2008). Cancer cells have a characteristic of high energy demand. Thus, inducing apoptosis through targeting the mitochondria seems to be a reasonable therapeutic strategy.

In recent years, substantial researches have shown that most commercially available antitumor drugs are derived from nature (Cragg and Newman, 2005; Newman and Cragg, 2012). Lycorine, a naturally occurring compound extracted from the *Amaryllidaceae* plant family, has been confirmed to exhibit antitumor activity in multiple cancer cell types (Lamoral-Theys et al., 2009; Cao et al., 2013; Zeng et al., 2017). Increasing evidence demonstrates that lycorine’s antitumor effects are mediated by inducing cell cycle arrest and antiproliferation, as evidenced in various cancer cell lines *in vitro* and in tumor xenograft models *in vivo* (Li et al., 2012; Cao et al., 2013; Wang et al., 2017). Drugs that target the apoptotic signalling pathway could potentially restore sensitivity to chemotherapy and cause cancer cell death (Gimenez-Bonafe et al., 2009; Fulda, 2015). Although it has been reported that lycorine induced mitochondrial dysfunction in multiple myeloma ARH-77 cells (Luo et al., 2015), the exact molecular mechanisms still remain unclear.

In the present study, we found that lycorine induced mitochondria-dependent apoptosis in hepatoblastoma HepG2 cells. Furthermore, an *in vivo* study revealed that lycorine induced apoptosis and inhibited tumor growth in a HepG2 xenograft mouse model. Meanwhile, all of these effects were in association with ROCK1 activation. Therefore, these findings may provide novel insights into the application of lycorine in the therapy strategies of hepatoblastoma.

## Materials and Methods

### Cells and Antibodies

Lycorine (A0415) was purchased from Must Biological Technology Co., Ltd (Chengdu, China). Y-27632 (S1049) was obtained from Selleckchem. Cyclosporine A (CSA, HY-B0579) was purchased from Medchem Express. Antibodies against cytochrome c were from Santa Cruz Biotechnology (sc-13156, Santa Cruz, CA, USA); β-actin was from Sigma-Aldrich (A5441, St. Louis, MO, USA); ROCK1 was from Abcam (ab25171, Burlingame, CA, USA); cleaved PARP (C-PARP, 5625), cleaved caspase 3 (C-Caspase 3, 9661), and Cox IV (4850) were from Cell Signaling Technology (Beverly, MA, USA).

### Cell Culture

HepG2 cells, SMMC7721 cells and BEL7402 cells were obtained from American Type Culture Collection (Manassas, VA, United States). Cells were cultured in Dulbecco’s modified Eagle’s medium (DMEM) supplemented with 10% fetal bovine serum. Cells were cultured in the presence of 5% CO_2_ at 37°C in humidified chambers.

### Cell Viability Assay

Cells (1 × 10^4^ cells/well) were seeded into a 96-well plate and incubated at 37°C with 5% CO_2_ overnight. Various concentrations of lycorine (0, 0.2, 0.5, 1, 2, 10, 20, 50, 100 μM) were added into each well the next day. After 48 h of incubation, 10 μl Cell Counting Kit 8 (CCK8, Dojindo, Japan) was added and cells were incubated for another 2 h. Viable cell numbers were valued by measurement of optical density (OD) at 450 nm with a microplate reader (Thermo, Flash, Waltham, MA, United States). The cell viability percentage was calculated as: viability percentage (%) = 100% × (absorption value of treatment group)/(absorption value of control group). All experiments were performed in triplicate.

### Flow Cytometry Assay

Cell apoptosis was measured using flow cytometry assay. After incubated with lycorine for 48 h, cells were collected and washed twice with cold PBS. Cells resuspended in 1× binding buffer were stained with FITC-annexin V and propidium iodide (PI) (BD, Biosciences, 556547). After incubation for 15 min in the dark, 200 µl 1× binding buffer was added and cells were analyzed using flow cytometry (FACScan, Becton Dickinson).

### ATP Luminescence Assay

The cellular ATP levels were detected using a firefly luciferase-based ATP determination kit (Beyotime, S0026). Briefly, after incubated with lycorine for 48 h, cells were lysed and centrifuged, and the ATP detection working solution was added to the supernatant later. The relative ATP levels were reflected as the percentage of levels that were observed using a microplate reader (Thermo, Varioskan Flash).

### Measurement of Mitochondrial Membrane Potential

The JC-1 kit (Beyotime, C2006) was used to measure the mitochondrial membrane potential (MMP). Cells were seeded in a 24-well plate or 96-well plate overnight, and later incubated with lycorine for 48 h. Cells were incubated with JC-1 reagent solution for 15 min at 37°C in the dark. Subsequently, cells were washed twice with JC-1 buffer solution. The fluorescence was observed using a fluorescence microscope (CKX31 OLYMPUS, Japan) or analyzed by a microplate reader (Thermo, Varioskan Flash) at 530 nm (green) and 590 nm (red).

### Mitochondrial Permeability Transition Pore (mPTP) Opening Detection Assay

Cells were seeded in a 96-well plate and incubated with lycorine for 48 h. After that, cells were incubated with 5 µM calcein-AM and 0.5 mM CoCl_2_ (cytosolic calcein quencher) for 15 min at 37°C. Then cells were washed twice with PBS and analyzed using a microplate reader (Thermo, Varioskan Flash) at an excitation wavelength of 488 nm and an emission wavelength of 525 nm.

### Western Blot Assay

Cells were harvested and washed twice with cold PBS. Then, cells were pelleted and lysed using RIPA buffer. Mitochondria were extracted from cells according to the manufacturer’s protocol (Beyotime, C3601). The protein concentrations were determined using a BCA protein assay kit (Beyotime, P0010); 15–60 μg of sample protein was separated by SDS-PAGE and transferred to PVDF membranes (Millipore, MA, USA). The membranes were blocked with 5% fat-free dry milk for 2 h. After washing with TBS-T for three times, membranes were cultured with primary antibodies at 4°C overnight. The following day, membranes were washed with TBS-T for three times after incubation with horseradish peroxidase-conjugated secondary antibodies at room temperature for 2 h. Protein bands were visualized using ECL agents according to the manufacturer’s instructions (Millipore, MA, USA).

### Immunoﬂuorescence

Cells were seeded in a 24-well plate and incubated with lycorine for 48 h. Cells were fixed with 4% paraformaldehyde for 15 min, then permeabilized with 0.1% Triton X-100 for 5 min and blocked with 5% fat-free dry milk for 30 min. The cells were subsequently co-incubated with ANT-1 and Cyp-D at 4°C overnight. Cells were counterstained with DAPI (C1005, Beyotime) followed by incubation with Alexa Fluor 488 donkey anti-rabbit IgG (A1101, 1:300) and Alexa Fluor 594 donkey anti-mouse IgG (A31573, 1:300) at 37°C for 1 h the following day. Images were taken using LSM780 confocal laser scanning microscope (Zeiss, Germany).

### Tumor Xenografts

Animal experiments were approved by the Army Medical University Institutional Animal Care. Nude mice (4 weeks old) were purchased form Vital River Laboratories (VRL, Beijing, China). HepG2 cells (2 × 10^6^ cells/mouse) were resuspended in the mixture of serum-free DMEM and Matrigel and injected subcutaneously into the right flanks of each mouse. Mice were randomly classified into two groups (*n* = 5 per group). Three days after tumor inoculation, the treatment group received lycorine (5 mg/kg/day, intraperitoneally for 3 weeks), and the control group received an equal volume of vehicle (saline). In the following days, tumor size and mice body weights were measured each 7 days; tumor volume was determined by a vernier caliper and calculated by the formula: volume = (width^2^ × length)/2. All animals were sacrificed after 3 weeks of drug exposure. Tumor tissues from representative mice were lysed and subjected to Western blot analysis and further examined by immunohistochemical analysis and hematoxylin and eosin (H&E) staining.

### Statistical Analysis

Data are expressed as the mean ± SD. The statistical analysis was performed by Student’s *t*-tests or one-way analysis of variance (ANOVA) with Tukey or Dunnett’s test using GraphPad Prism 6.0 statistical analysis software. *P < 0.05, **P < 0.01, and ***P < 0.001 were considered as statistical significance.

## Results

### Lycorine Induced Apoptosis in HepG2 Cells

**Figure 1:**
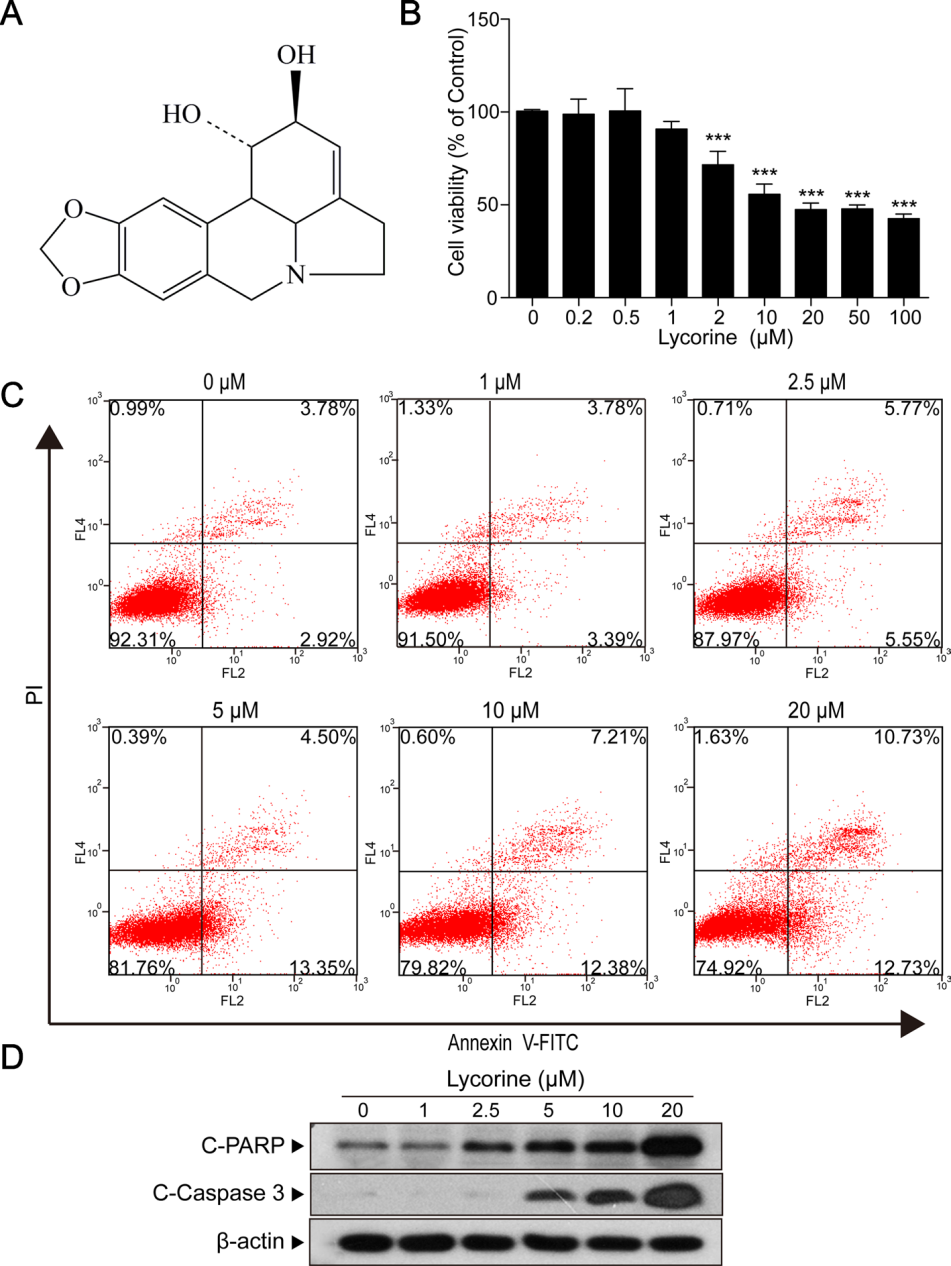
Lycorine induced apoptosis in HepG2 cells. **(A)** The chemical structure of lycorine. **(B)** HepG2 cells were treated with various concentrations of lycorine (0, 0.2, 0.5, 1, 2, 10, 20, 50, and 100 μM) for 48 h, cell viability was measured using the CCK8 assay. **(C)** Cells were treated with various concentrations of lycorine (0, 1, 2.5, 5, 10, and 20 μM) for 48 h. Apoptosis was determined by annexin V-FITC/PI staining. **(D)** Western blot assay was used to examine the levels of cleaved PARP (C-PARP) and cleaved caspase 3 (C-Caspase 3). Data are presented as the means ± S.D. (n = 3). ***P < 0.001 compared to control.

The chemical structure of lycorine is shown in **Figure 1A**. First, we performed CCK8 assay to assess the effects of lycorine on cytotoxicity in HepG2 cells, SMMC7721 cells, and BEL7402 cells. As shown in **Supplementary Figure 1**, exposure of HepG2 cells, SMMC7721 cells, and BEL7402 cells to lycorine (0, 2, 20, 50, and 100 μM) for 24 h resulted in a significant inhibition of cell viability to different degrees. Among them, HepG2 cells were most sensitive to lycorine’s exposure. Thus, HepG2 cells were chosen for the following experiments.

Next, HepG2 cells incubated with 20 μM lycorine were exposed to different time intervals (0, 6, 9, 12, 24, 36, and 48 h) and we found that lycorine induced cell viability inhibition in a time-dependent manner (**Supplementary Figure 2**; *P < 0.05, ***P < 0.001). Furthermore, our results also indicated that cells treated with various concentrations of lycorine (0, 0.2, 0.5, 1, 2, 10, 20, 50, and 100 μM) for 48 h resulted in a significant decrease in cell viability in a dose-dependent manner (**Figure 1B**; ***P < 0.001). Meanwhile, our flow cytometry analysis revealed that exposure of cells to lycorine increased the apoptosis rate in a concentration-dependent manner (**Figure 1C**). Consistent with these findings, Western blot analysis revealed that lycorine treatment resulted in apoptosis related proteins cleaved PARP (C-PARP) and cleaved caspase 3 (C-Caspase 3) accumulation in HepG2 cells (**Figure 1D**). All of these results suggested that lycorine induced apoptosis in HepG2 cells.

**Figure 2:**
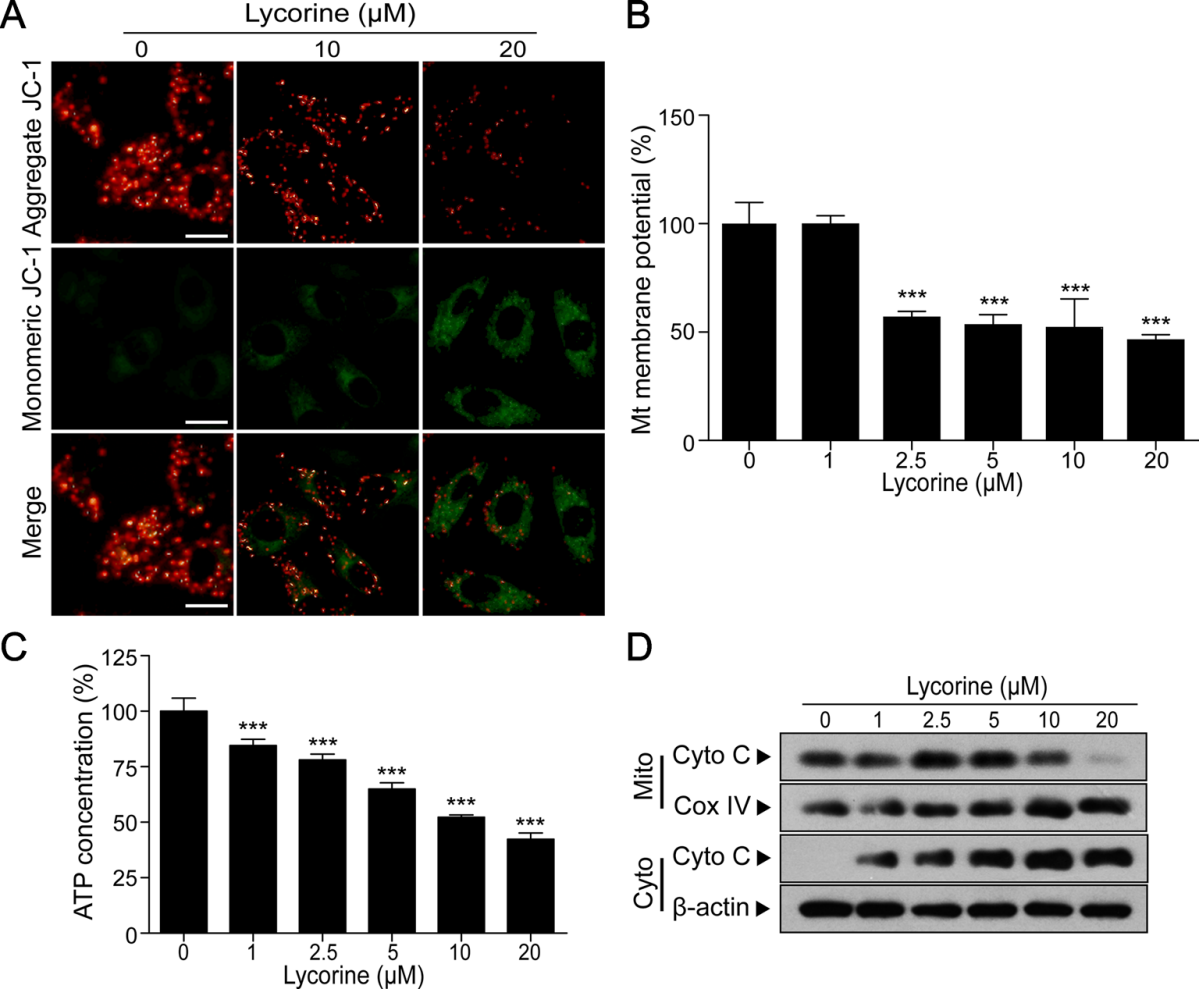
Lycorine induced mitochondrial apoptosis in HepG2 cells. **(A)** HepG2 cells were treated with lycorine (0, 10, 20 μM) for 48 h, the mitochondrial membrane potential (MMP) was analyzed with JC-1 staining using fluorescence microscopy. Scale bar: 50 μm. **(B)** MMP was measured by JC-1 staining assay and analyzed using microplate reader. **(C)** Cells were treated with various concentrations of lycorine (0, 1, 2.5, 5, 10, and 20 μM) for 48 h; an ATP determination kit was used to determine the cellular ATP levels. **(D)** Cells were treated with various concentrations of lycorine (0, 1, 2.5, 5, 10, and 20 μM) for 48 h; mitochondrial (Mito) and cytosolic (Cyto) fractions were prepared. The levels of cytochrome c (Cyto C) in Mito and Cyto were determined using Western blot assay. Data are presented as the means ± S.D. (n = 3). ***P < 0.001 compared to control.

### Lycorine Induced Mitochondrial Apoptosis in HepG2 Cells

Previous studies have reported that mitochondrial pathway plays a critical role in apoptosis and the loss of MMP is reported to in association with mitochondrial injury (Pokorny et al., 2014; Fulda, 2015). To further investigate whether lycorine induces HepG2 cells apoptosis through a mitochondria-dependent pathway, we detected the changes of MMP under lycorine treatment. JC-1, a mitochondrial MMP-sensitive dye, is often used to detect the changes in MMP. After staining with JC-1, untreated cells have strong red fluorescence (JC-1 aggregation) and weak green florescence (JC-1 monomer). Accompanied by the loss of MMP, JC-1 aggregation dissipates to monomers, leading to a shift from red to green. As shown in **Figure 2A**, lycorine treatment (0, 10, and 20 μM) resulted in a decrease in red fluorescence and an increase in green fluorescence, suggesting that lycorine resulted in the loss of MMP. Furthermore, we also found that lycorine treatment resulted in a significant decrease in the levels of MMP in a dose-dependent manner using a microplate reader (**Figure 2B**; ***P < 0.001). It has been reported that mitochondrial dysfunction is usually followed by ATP depletion (Skulachev, 1999; Singleterry et al., 2014). As shown in **Figure 2C**, we found that there was a significant decrease in the ATP levels under lycorine treatment (***P < 0.001). It is well known that during intracellular apoptosis, the loss of Δψm is usually accompanied by cytochrome c (Cyto C) release from the mitochondria into the cytosol (Srinivasan and Avadhani, 2012). Our Western blot analysis also revealed that lycorine promoted Cyto C release from the mitochondria into the cytosol (**Figure 2D**). Collectively, these findings indicated that lycorine induced mitochondrial apoptosis in HepG2 cells.

### Lycorine Induced mPTP Opening in HepG2 Cells

**Figure 3:**
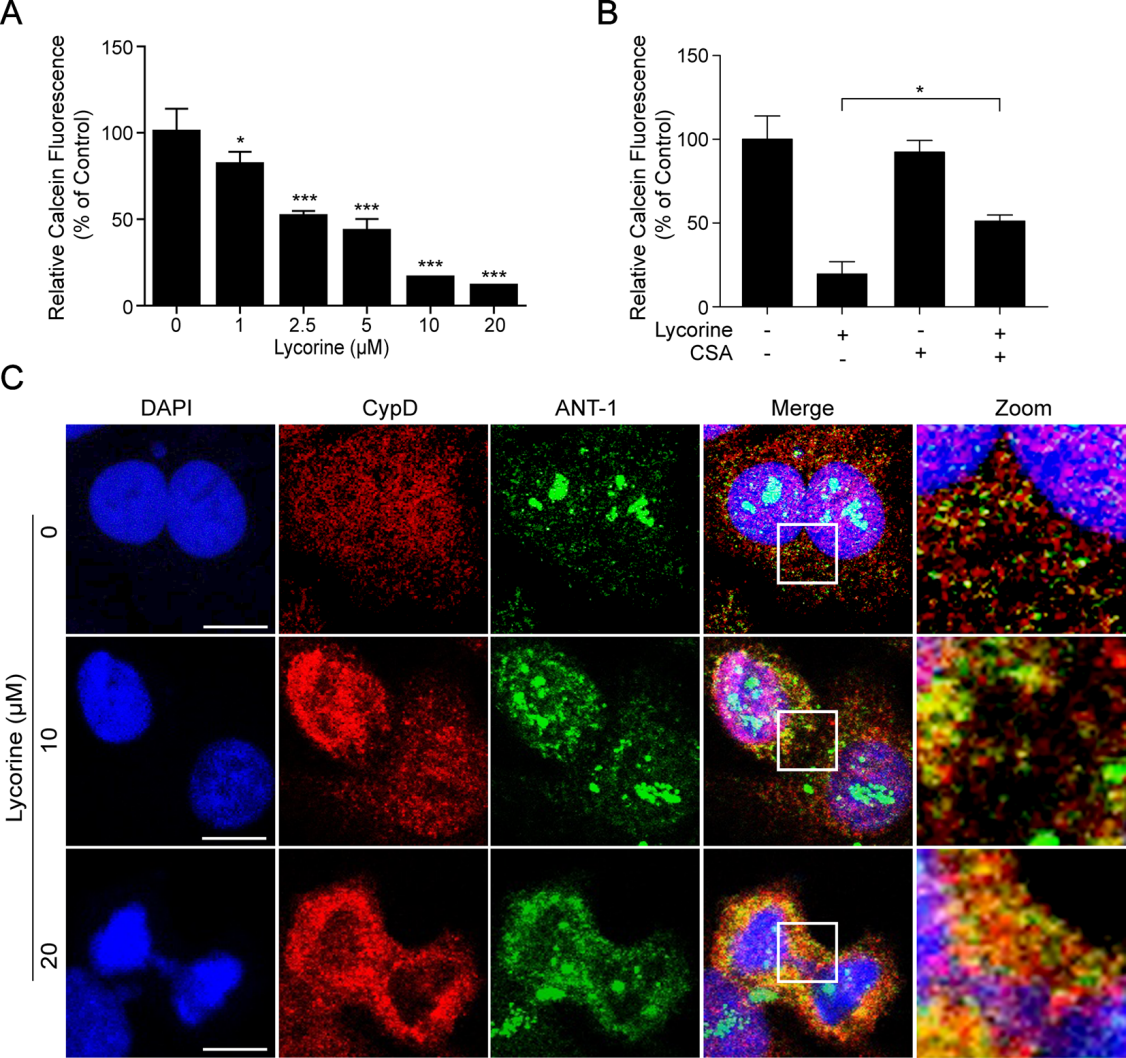
Lycorine induced mPTP opening in HepG2 cells. **(A)** After incubated with lycorine (0, 1, 2.5, 5, 10, and 20 μM) for 48 h, HepG2 cells were stained with calcein-AM and CoCl_2_; calcein fluorescence was detected using microplate reader. **(B)** After preincubated with cyclosporine A (CSA, 5 µM) for 2 h, cells were treated with lycorine (20 μM) for 48 h, the calcein fluorescence was detected using microplate reader. **(C)** Cells were counterstained with CypD, ANT-1, and DAPI. Images were captured using confocal microscope. Scale bar: 10 μm. *P < 0.05 and ***P < 0.001 compared to control.

It is well-known that mitochondrial permeability transition pore (mPTP) opening plays an important role in mitochondria-induced pro-death function and eventually leads to cell apoptosis (Halestrap et al., 2002; Halestrap, 2006; Baines, 2009). In the present study, we performed calcein-AM staining combined with CoCl_2_ to explore whether mPTP opening occurred in lycorine-induced mitochondrial apoptosis. Calcein AM, a colourless esterase substrate, possesses the ability to enter living cell membranes and form a very polar green fluorescent material. Then the fluorescence from cytosolic calcein is quenched by CoCl_2_, and a fluorescence detector is used to detect the intensity of fluorescence and estimate the opening degree of mPTP when the fluorescence from the mitochondrial calcein is maintained. As shown in **Figure 3A**, lycorine treatment resulted in a significant reduction of relative calcein fluorescence (*P < 0.05, ***P < 0.001), and this can be blocked by cyclosporine A (CSA), a well-known inhibitor of mPTP (**Figure 3B**; *P < 0.05). Studies have reported that mPTP is composed of at least three major proteins: voltage-dependent anion channel (VDAC) located on the outer mitochondrial membrane (OMM), adenine nucleotide translocator (ANT) located on the inner mitochondrial membrane (IMM), and cyclophilin D (CypD) located in the mitochondrial matrix. It has been reported that the binding of CypD and ANT-1 initiates the opening of mPTP (Juhaszova et al., 2008; Zhen et al., 2014). Therefore, we examined the effect of lycorine on the interaction between CypD and ANT- 1. As shown in **Figure 3C**, our results indicated that lycorine treatment resulted in an increase in the association between CypD and ANT-1. Taken together, these findings suggested that lycorine induced mPTP opening in HepG2 cells.

### ROCK1 Activation Played a Significant Role in Lycorine-Induced Apoptosis

**Figure 4:**
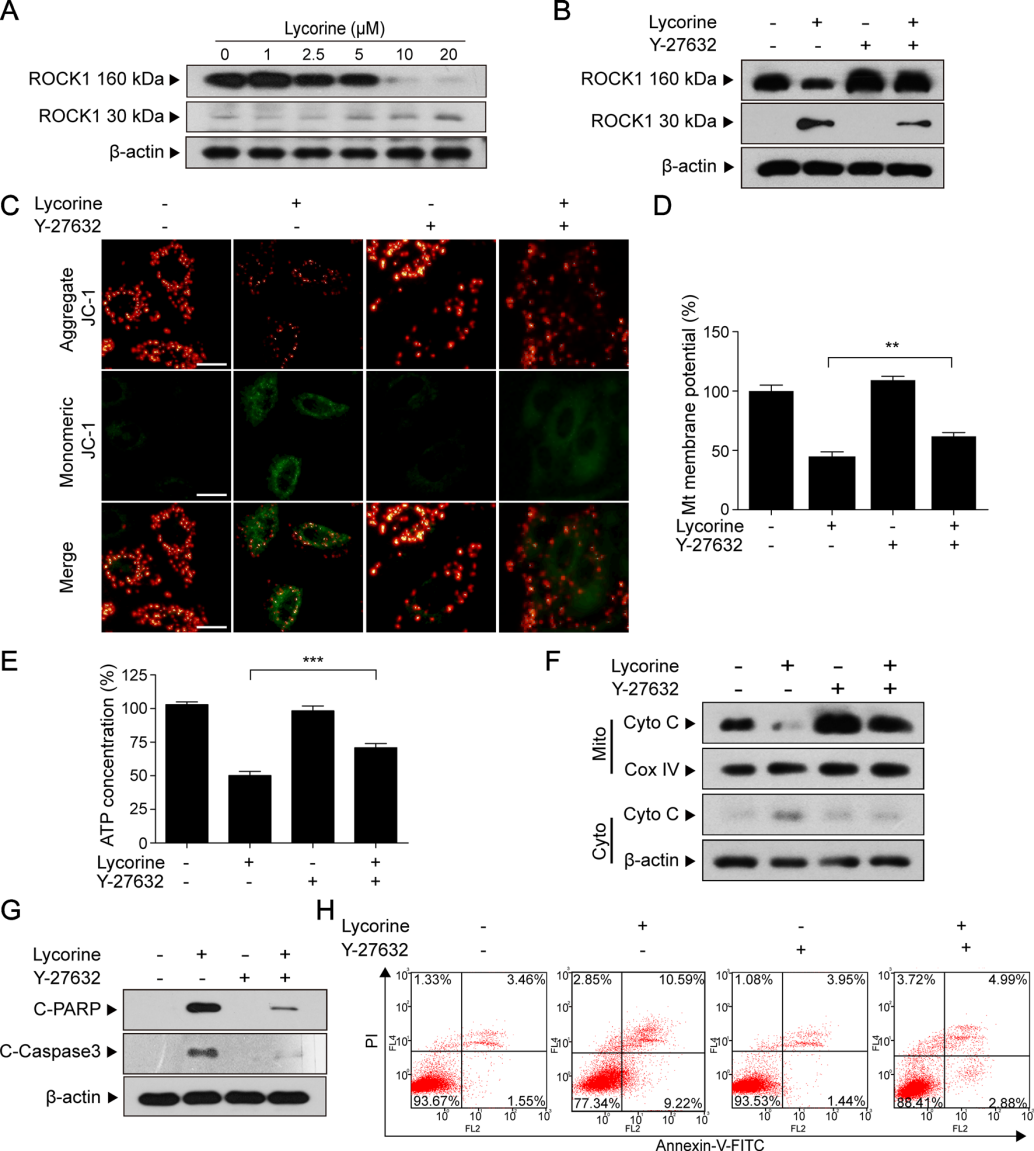
ROCK1 activation played a significant role in lycorine-induced apoptosis. **(A)** HepG2 cells were treated with lycorine (0, 1, 2.5, 5, 10, and 20 µM) for 48 h; Western blot assay was performed to detect the levels of ROCK1 (160 kDa) and cleaved ROCK1 (30 kDa). **(B)** After pre-incubation with Y-27632 (20 µM) for 2 h, cells were incubated with lycorine (20 µM) for 48 h, the expression of ROCK1 (160 kDa) and cleaved ROCK1 (30 kDa) were detected using Western blot assay. **(C** and **D)** JC-1 staining was used to determine the changes of MMP; images were captured using confocal microscope. Otherwise, the fluorescence was detected using microplate reader. **(E)** ATP levels were detected using an ATP determination kit. **(F)** Western blot assay was used to examine the levels of Cyto C in mitochondria (Mito) and cytosol (Cyto). **(G)** Western blot assay was used to determine the expression of C-Caspase 3 and C-PARP. **(H)** Cells were stained with annexin V-FITC/PI, and flow cytometry was used to determine the percentage of apoptotic cells. **P < 0.01 and ***P < 0.001 compared to control.

Previous studies have shown that ROCK1 plays a major role in a variety of cellular activities (Sebbagh et al., 2001; Zheng et al., 2011). Furthermore, ROCK1 activity is involved in cytoskeletal reorganization and membrane blebbing during apoptosis. To determine whether ROCK1 activation is involved in lycorine-induced apoptosis, the ROCK1 expression was detected using Western blot assay. Our results revealed that HepG2 cells treated with various concentrations of lycorine (0, 1, 2.5, 5, 10 and 20 μM) for 48 h exhibited decreased expression of ROCK1 (160 kDa) and increased cleaved ROCK1 (30 kDa) in a concentration-dependent manner (**Figure 4A**). To further assess the role of ROCK1 in lycorine-induced apoptosis, we employed Y-27632, a ROCK inhibitor. Western blot analysis showed that pre-incubation with Y-27632 partly blocked lycorine-induced ROCK1 activation (**Figure 4B**). JC-1 staining showed that co-treatment with Y-27632 significantly attenuated lycorine-induced MMP loss (**Figure 4C, D**; **P < 0.01). Cells pre-incubated with Y-27632 also attenuated lycorine-induced ATP depletion and Cyto C release (**Figure 4E, F**; ***P < 0.001). Moreover, the administration of Y-27632 significantly attenuated lycorine-mediated caspase activation and PARP cleavage (**Figure 4G**). Flow cytometry demonstrated that pre-incubation with Y-27632 attenuated lycorine-induced cell apoptosis (**Figure 4H**). Taken together, these findings demonstrated that ROCK1 activation played a significant role in lycorine-induced apoptosis.

### Lycorine Inhibited Tumor Growth, Induced Apoptosis, and Activated ROCK1 in a HepG2 Xenograft Model

**Figure 5:**
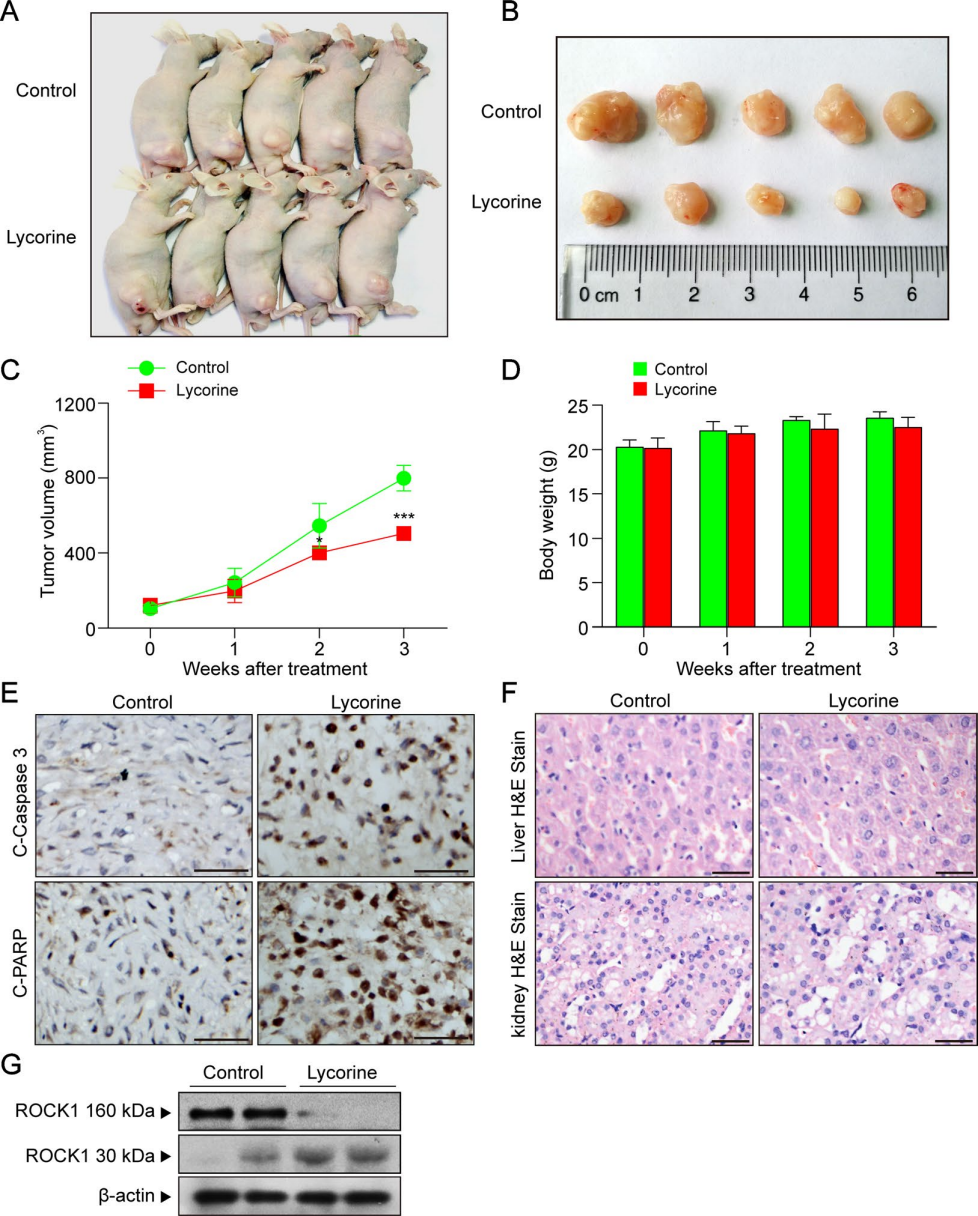
Lycorine inhibited tumor growth, induced apoptosis, and activated ROCK1 in a HepG2 xenograft model. **(A)** Representative images of mice in each group. **(B)** Representative images of each tumor in each group. **(C)** Tumor growth curve for vehicle control and lycorine-treated mice (**P < 0.01 vs the control group). **(D)** Body weight curve of vehicle control and lycorine-treated mice. **(E)** Representative tumor tissues were fixed and subjected to immunohistochemistry staining for C-Caspase 3 and C-PARP. **(F)** Representative H&E-stained liver and kidney sections in vehicle control and lycorine-treated mice. Scale bars: 25 μm. **(G)** Western blot assay from representative tumor tissues of vehicle control and lycorine-treated mice detected the expression of ROCK1 (160 kDa) and cleaved ROCK1 (30 kDa) (*P < 0.05 and ***P < 0.001 compared to control).

To determine whether lycorine exhibits antitumor activity *in vivo*, nude mice were inoculated with xenografts (**Figure 5A, B**). Our results revealed that lycorine treatment resulted in a significant suppression of tumor growth (**Figure 5C**; *P < 0.05, ***P < 0.001). However, there was no statistically significant changes in body weight (**Figure 5D**) or other signs of toxicity like impaired movement and posture, indigestion, diarrhea, areas of redness, swelling, or agitation when compared to control group. Furthermore, immunohistochemical analysis revealed that lycorine caused an increase in immunoreactivity for C-Caspase 3 and C-PARP (**Figure 5E**). Otherwise, liver and kidney samples were also excised, sectioned, and analyzed by hematoxylin and eosin (H&E) staining. Our results showed that there is no morphological difference between lycorine-treated and control groups (**Figure 5F**). To determine whether ROCK1 activation is involved in lycorine-induced apoptosis *in vivo*, Western blot assay was performed. Our results showed that lycorine induced ROCK1 activation in the HepG2 xenograft model (**Figure 5G**). Taken together, these findings demonstrated that lycorine inhibited tumor growth and induced apoptosis in a HepG2 xenograft mouse model in association with ROCK1 activation.

## Discussion

In the present study, we demonstrated that lycorine inhibited tumor growth of HepG2 xenografts *in vivo* and induced apoptosis in hepatoblastoma HepG2 cells through a mitochondria-dependent pathway. Furthermore, we found that ROCK1 activation plays a critical role in lycorine-induced mitochondrial apoptosis. Our study may provide a mechanistic basis for the therapeutic rationale to develop lycorine as a novel drug candidate to treat hepatoblastoma.

Inefficient apoptosis is a proverbial hallmark of cancer cells. Thus, it is of great significance for us to examine apoptotic signaling pathways to explore novel strategies for cancer treatment. In recent years, most studies focus on elucidating signaling pathways involved in lycorine-induced apoptosis. For instance, lycorine was shown to induce apoptosis in A549 cells *via* AMPK-mammalian targeting of the rapamycin (mTOR)-S6K signaling pathway (Zeng et al., 2017). Lycorine inhibits breast cancer growth and metastasis by inducing apoptosis and blocking the Src/FAK-involved pathway (Ying et al., 2017). Furthermore, it has been reported that lycorine promoted autophagy and apoptosis *via* TCRP1/Akt/mTOR axis inactivation in human hepatocellular carcinoma (Yu et al., 2017). Mitochondria, an important regulatory organelle in the intrinsic apoptotic pathway, play a critical role in the cell apoptosis process. Cancer cells characteristically proliferate in a rapid manner and maintain viability under potentially toxic conditions. Thus, inducing apoptosis in cancer cells through a mitochondria-dependent pathway appears to be an effective strategy to alter metabolic efficiencies and mitochondrial function. In our study, we found that lycorine induced mitochondiral apoptosis by mPTP opening with the following evidence. First, lycorine induced apoptosis, PAPR cleavage and caspase activation in HepG2 cells and HepG2 xenograft mouse model. Second, lycorine decreased MMP in HepG2 cells in a dose-dependent manner. Moreover, lycorine facilitated ATP depletion and promoted cytochrome c (Cyto C) release from mitochondria into cytosol. Third, lycorine increased the association between CypD and ANT-1, resulted in mPTP opening and Ca^2+^ release. Otherwise, pre-incubation cells with cyclosporine A (CSA), an mPTP opening inhibitor, significantly blocked lycorine-induced mPTP opening.

Rho-associated coiled coil-containing protein kinase (ROCK) is a key downstream effector of the small GTPase RhoA proteins. ROCK1, one isoform of ROCK (ROCK1 and ROCK2), is necessary for membrane blebbing and cytoskeletal reorganization during apoptosis (Coleman et al., 2001). In recent years, ROCK1-targeting treatment strategy has drawn much attention for its promising clinical application in cancer therapy, diabetes, stem cell biology, and so on (Chun et al., 2011; Castro et al., 2013). Moreover, ROCK1 is reported to be cleaved and activated by a variety of mechanical stimuli and biochemical mediators in the regulation of apoptosis, as evidenced in various cell lines and animal disease models (Liu et al., 2013; Zhang et al., 2016). In our study, we found ROCK activation was involved in lycorine-induced apoptosis based on the following evidence. First, lycorine treatment decreased the levels of ROCK1 (160 kDa) and increased the cleaved ROCK1 (30 kDa). Second, cells pre-incubated with Y-27632, a ROCK inhibitor which was widely used to evaluate the role of ROCK kinases in a variety of cells and animal models (Ohki et al., 2001; Sinnett-Smith et al., 2001), markedly blocked lycorine-induced mitochondrial injury and cell apoptosis. In addition, our *in vivo* studies revealed that lycorine significantly inhibited tumor growth in HepG2 xenografts in association with ROCK1 activation. Recently, it has been reported that lycorine represses Akt/mTOR signalling *via* decreasing the levels of TCRP1 protein, resulting in apoptotic and autophagic processes activation (Yu et al., 2017). Moreover, recent studies have reported that ROCK1 is an upstream regulatory molecule of Akt/mTOR signalling (Vo et al., 2011; Zheng et al., 2018). Our research further clarified the anti-tumor mechanism of lycorine, suggesting that lycorine-mediated ROCK1 activation and mitochondria-dependent apoptosis may be a novel therapeutic strategy for hepatoblastoma treatment.

## Conclusion

**Figure 6:**
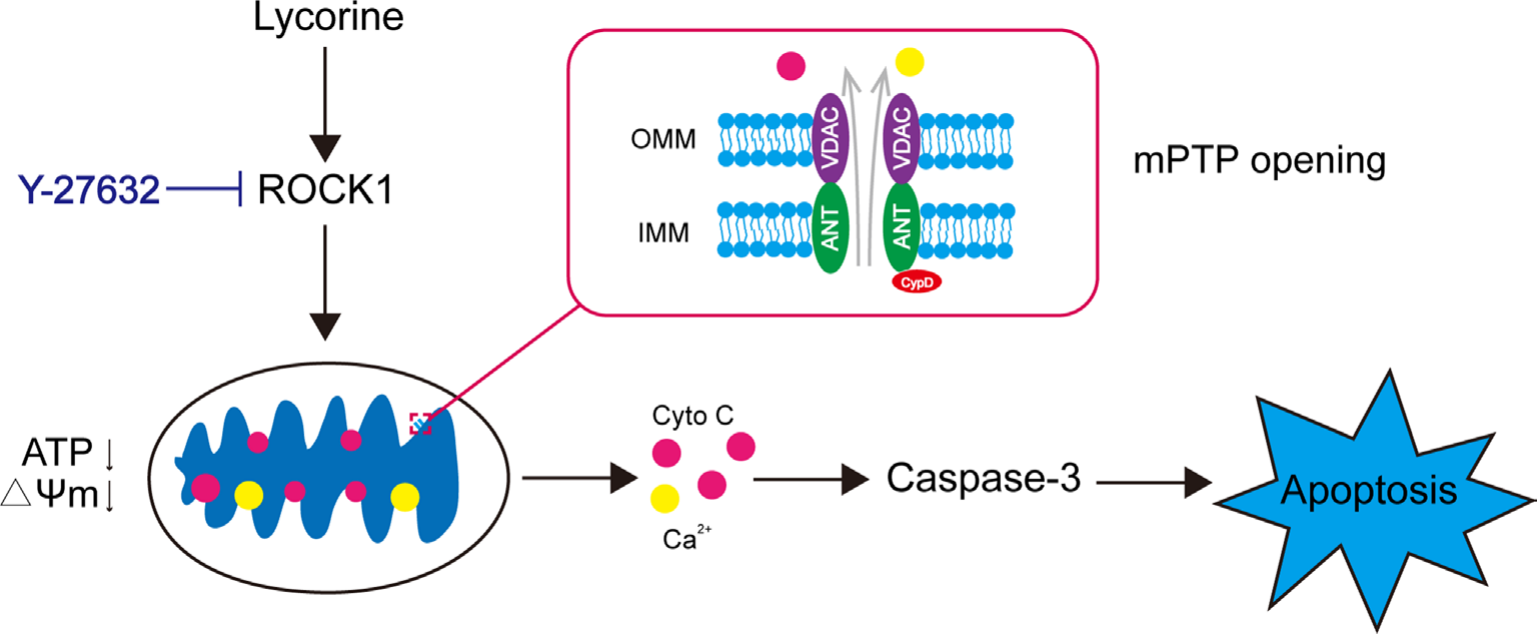
Model of lycorine-induced mitochondrial apoptosis in HepG2 cells. Lycorine induces ROCK1 cleavage/activation, leading to mPTP opening, ATP depletion, the loss of MMP, Ca^2+^, and Cyto C release. Caspases are activated, which eventually results in apoptosis. Y-27632, a ROCK specific inhibitor, partly block lycorine-induced ROCK1 activation, mitochondrial injury and HepG2 cell apoptosis.

In conclusion, our findings indicated that lycorine induced mitochondrial apoptosis of HepG2 cells *in vitro* and inhibited tumor growth in a HepG2 xenograft mouse model *in vivo*. Collectively, these findings suggest a hierarchy of events in lycorine-induced apoptosis in which ROCK1 activation represents the primary insult, leading to mPTP opeining, Ca^2+^ release, promoting Cyto C released from mitochondria to cytosol, caspase activation and eventually resulted in apoptosis (**Figure 6**). All of these results may provide a novel mechanistic basis for the application of lycorine in hepatoblastoma treatment.

## Ethics Statement

The university’s institutional animal care and use committee approved all the animal studies.

## Author Contributions

W-yL, QT, G-bL and RZ designed the research. W-yL, QT, QZ, C-pH, and F-fS performed the experiment. J-bH, Y-lL, MZ, and W-jL analyzed the data. W-yL, G-bL, and RZ wrote the paper.

## Funding

This work was supported by the National Natural Science Foundation of China (grant nos. 81801273 and 81874357), Clinical Research Projects of the Second Affiliated Hospital, Army Medical University (2016YLC12), and Natural Science Foundation of Chongqing (cstc2018jcyjAX0183).

## Conflict of Interest Statement

The authors declare that the research was conducted in the absence of any commercial of financial relationships that could be construed as a potential conflict of interest.
