# Supplementary material for: Lycorine Induces Mitochondria-Dependent Apoptosis in Hepatoblastoma HepG2 Cells Through ROCK1 Activation
Source: Front Pharmacol. 2019 Jun 6;10:651. doi: 10.3389/fphar.2019.00651 (PMC6589644; doi:10.3389/fphar.2019.00651)
Supplement: Supplementary file 1 [file Table_1.doc]

**Supplementary Information**

**Lycorine Induces Mitochondria-Dependent Apoptosis in Hepatoblastoma HepG2 cells Through ROCK1 Activation**

*Wu-yi Liu^†^, Qin Tang^†^, Qian Zhang, Chang-peng Hu, Jing-bin Huang, Fang-fang Sheng, Ya-li Liu, Min Zhou, Wen-jing Lai, Guo-bing Li* and Rong Zhang**

*Department of Pharmacy,* *The Second Affiliated Hospital, Army Medical University, Chongqing, China*

**Supplementary Figure 1**

**
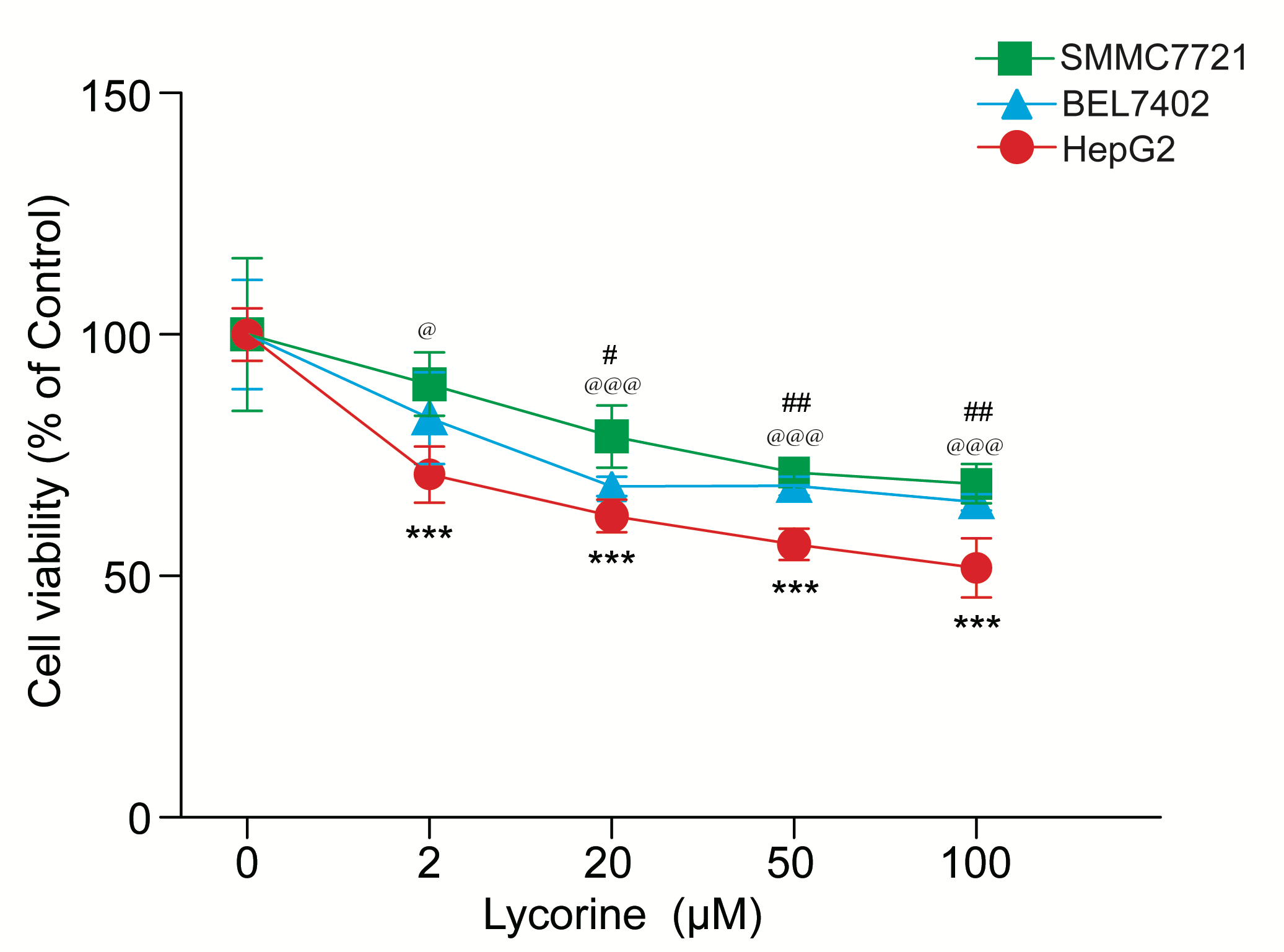
**

**Supplementary Figure 1.** Lycorine inhibited cell viability of SMMC7721 cells, BEL7402 cells and HepG2 cells. Cell viability was determined using CCK8 assay after SMMC7721 cells, BEL7402 cells and HepG2 cells were exposure to various concentrations of lycorine (0, 2, 20, 50, 100 μM) for 24 h. Data are presented as the means ± S.D. (n=3). ^#^*P* < 0.05 and ^##^*P* < 0.01 compared to control 0 h-group of SMMC7721 cells. @*P* < 0.05 and @@@*P* < 0.001 compared to control 0 h-group of BEL7402 cells; ****P* < 0.001 compared to control 0 h-group of HepG2 cells.

**Supplementary Figure 2**

**
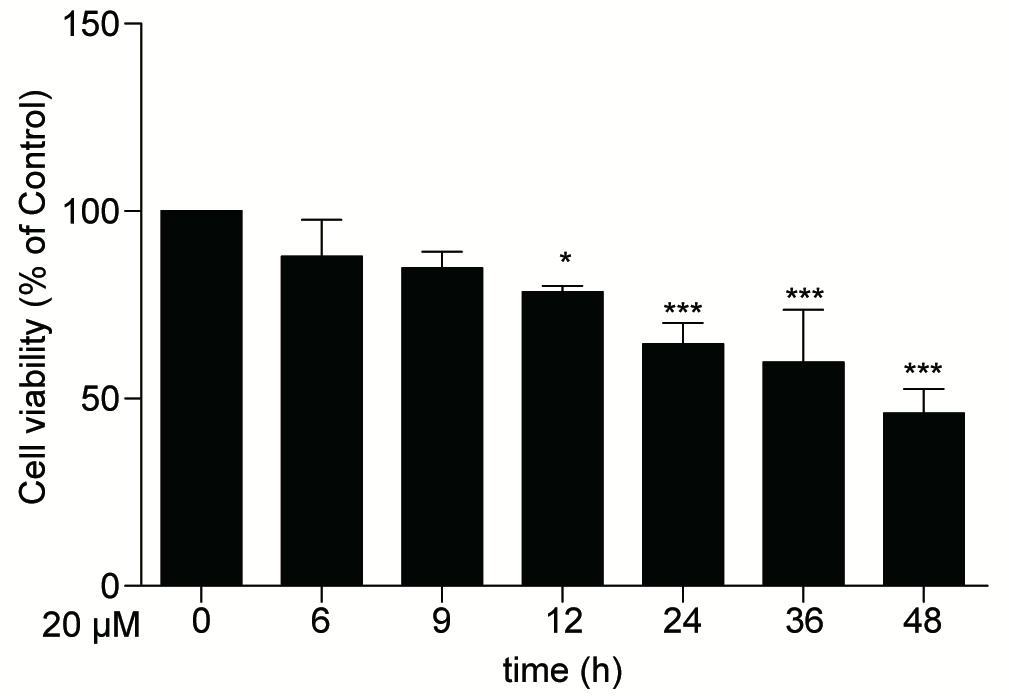
**

**Supplementary Figure 2.** Viability of HepG2 cells treated with lycorine for different intervals by CCK8 assay. Cell viability was detected using CCK8 assay after HepG2 cells exposed to lycorine (20 μM) for 0, 6, 9, 12, 24, 36, 48 h. Data are presented as the means ± S.D. (n=3). **P* < 0.05 and ****P* < 0.001 compared to control.
